# Women’s attitudes, beliefs and values about tests, and management for hypertensive disorders of pregnancy

**DOI:** 10.1186/s12884-021-04144-2

**Published:** 2021-09-30

**Authors:** Shenaz Ahmed, Alina Brewer, Eleni Z. Tsigas, Caryn Rogers, Lucy Chappell, Jenny Hewison

**Affiliations:** 1 Leeds Institute of Health Sciences, School of Medicine, Worsley Building, University of Leeds, Leeds, LS2 9NL UK; 2 Preeclampsia Foundation, Predictive Laboratories, Inc., Salt Lake City, UT USA; 3 Preeclampsia Foundation, Melbourne, FL USA; 4 Preeclampsia Foundation, Pittsburgh, PA USA; 5 School of Life Course Science, King’s College London, London, UK; 6 Leeds Institute of Health Sciences, University of Leeds, Leeds, UK

**Keywords:** Hypertension, Preeclampsia, Decision-making, Prevention, Preventive, Risk management, Aspirin

## Abstract

**Background:**

Advances in research suggest the possibility of improving routine clinical care for preeclampsia using screening (predictive) and diagnostic tests. The views of women should be incorporated into the way in which such tests are used. Therefore, we explored the views of women with experience of preeclampsia and other hypertensive disorders in pregnancy (HDPs) about predictive and diagnostic tests, treatment risks, and expectant management.

**Method:**

Eight hundred and seven women with experience of preeclampsia or other HDPs completed an online questionnaire. These women were participants in the Preeclampsia Registry (USA). The questionnaire contained 22 items to elicit women’s views about predictive tests (*n* = 8); diagnostic tests (*n* = 5); treatment risks (*n* = 7), and expectant management (*n* = 2). An optional text box allowed participants to add qualitative open-ended comments. Levels of agreement with the statements were reported descriptively for the sample as a whole, and a preliminary investigation of the role of lived experience in shaping women’s views was conducted by comparing subgroups within the sample based on time of HDP delivery (preterm/term). The qualitative data provided in the optional text box was analysed using inductive thematic analysis to examine participants’ responses.

**Results:**

Women generally favored predictive and diagnostic testing, although not because they would opt for termination of pregnancy. Participants generally disagreed that taking daily low-dose aspirin (LDA) would make them nervous, with disagreement significantly higher in the preterm delivery subgroup. A high proportion of participants, especially in the preterm delivery subgroup, would take LDA throughout pregnancy. The majority of participants would be more worried about the possibility of preeclampsia than about the risks of treatments to their health (60%), and that proportion was significantly higher in the preterm delivery subgroup. There were no differences between subgroups in the views expressed about expectant management, although opinion was divided in both groups. Overall, most participants opted to put the baby’s interests first.

**Conclusion:**

Women with experience of hypertensive disorders were enthusiastic about improved predictive and diagnostic tests. However, varied views about treatment options and expectant management suggest the need for a shared decision-making tool to enable healthcare professionals to support pregnant women’s decision-making to maximize the utility of these tests and interventions.

**Supplementary Information:**

The online version contains supplementary material available at 10.1186/s12884-021-04144-2.

## Background

Preeclampsia, a leading cause of maternal and fetal morbidity and mortality, causes approximately 42,000 deaths worldwide annually and complicates 2–8% of pregnancies [1, 2]. Characterised by hypertension and multi-organ dysfunction, preeclampsia can also be complicated by fetal growth restriction and preterm birth [3, 4]. Effective treatment for preeclampsia is delivery, regardless of gestational age, which will not always prevent or ameliorate postpartum preeclampsia [4, 5]. A history of preeclampsia raises risk of preeclampsia in subsequent pregnancies and risk of cardiovascular disease in subsequent years above that of women with normotensive pregnancy history [6]. Prophylactic low-dose aspirin (LDA) lowers the risk of developing preterm preeclampsia [7].

Although early identification of women at risk enables timely management [1], diagnosis of preeclampsia is reliant on blood pressure readings, quantification of proteinuria and bloodwork, and nonspecific symptoms (e.g. headache) indicative of severe features. Variation in onset, clinical presentation, and severity affect the specificity and reliability of clinical assessments, making screening and diagnosis challenging [1, 8].

Advances in research suggest the possibility of improving routine clinical care using screening (predictive) and diagnostic tests [9–12]. Such tests would be valuable for the management of the mother and baby, so may be implemented in the future. Maternal and fetal interests may conflict when pregnant women have to make earlier decisions about care. Implementation of these tests should be based on their acceptance by the target population. However, there is a paucity of literature on women’s views and decision aids on this topic.

Furthermore, although research suggests that women with hypertensive disorders beyond 37 weeks gestation would benefit from planned delivery [13], a March of Dimes public education campaign emphasizes that “39 weeks is worth the wait” to reduce elective inductions. These messages may affect women’s perceptions on the timing of delivery.

Resolving these dilemmas requires understanding women’s views about predictive and diagnostic tests, and how these affect decisions about treatment, and early delivery. There is an increasing focus on decision aids to guide communications and shared decision-making [14], and maternal-fetal health organizations’ education and support of women. This study explored the views of a convenience sample of women with experience of hypertensive disorders in pregnancy (HDPs) about tests, treatment risks, and expectant management to guide development of consumer messages and education campaigns, and inform future research.

## Methods

An online self-completion questionnaire was developed and piloted through multiple iterations by the Preeclampsia Foundation, clinicians, and social scientists. Questions addressed dilemmas and drew on phrasing advanced by women with histories of HDPs. Survey participants were informed that tests and treatments were not necessarily available to patients, “preeclampsia” was described to include most HDPs, and definitions of screening and diagnostic tests were provided. The questionnaire contained demographic and clinical history questions, and 22 items designed to elicit participants’ views about: predictive tests (*n* = 8), diagnostic tests (*n* = 5), treatment (*n* = 7), and expectant management (*n* = 2). To minimize acquiescence response bias, both positively and negatively framed questions were included [15]. Participants rated each item on a six-point Likert scale ranging from Strongly Disagree to Strongly Agree. An *optional* text box allowed participants to add comments. For the full questionnaire, please see Additional file 1.

### Participants and procedure

Data collection was carried out by the Preeclampsia Foundation, Florida, USA, via https://www.preeclampsiaregistry.org. Initially, Preeclampsia Registry members were recruited through Preeclampsia Foundation communications, web searches, and social media. Informed consent was obtained on-line from all members willing to participate, and they then completed a questionnaire to self-report health history, diagnosis of a hypertensive disorder, and pregnancy outcomes. (A previous Preeclampsia Registry study showed that 181 (94.3%) of 192 participants accurately recalled their pregnancy diagnosis [16].) Three thousand, five hundred and fifty eight women reporting a history of an HDP were invited by email to complete the Your Point of View questionnaire. Eight hundred and sixty one participants started the questionnaire and 807 completed it between October 2016 and July 2019. There were no significant differences in baseline demographics between women responding and those not responding (see Table 1). Data analysis was exploratory, investigating the views of a convenience sample of women to inform future research.

**Table 1:** Demographic information and pregnancy outcomes for participants (*n* = 807) and non-responders (*n* = 2697)

Self-Reported Characteristics	Participants n (%)	Non-Responders n (%)
**Overall**		
Race:		
Caucasian	771 (95.5)	2453 (91.0)
Black	18 (2.2)	81 (3.0)
Native American	13 (1.6)	63 (2.3)
Asian	6 (0.7)	63 (2.3)
Pacific Islander	4 (0.5)	8 (0.3)
Asian Indian	1 (0.1)	13 (0.5)
One or more preterm HDP deliveries (< 37 weeks)	545 (67.5)	1873 (69.4)
**At time of first HDP pregnancy**		
Maternal age at delivery (years), mean +/− SD	30.5+/−6.0	29.2+/−5.2
Years since pregnancy at start of survey	5.7+/−7.5	8.2+/− 7.3
Nulliparity	726 (90.0)	2441 (90.5)
Assisted Reproductive Technology	84 (10.4)	224 (8.3)
Started prenatal care- Less than 12 weeks	774 (95.9)	2284 (84.7)
Multiple gestation	32 (4.0)	106 (3.9)
Elevated liver enzymes	469 (58.1)	1202 (44.6)
Kidney problems	131 (16.2)	452 (16.8)
Low platelet count	357 (44.2)	945 (35)
Fluid in lungs	77 (9.5)	202 (7.5)
Seizure	49 (6.1)	113 (4.2)
Increased protein	579 (71.7)	1719 (63.7)
^a^Maximum systolic blood pressure (mm Hg), mean +/− SD	181+/−24.9	184+/− 26.7
^a^Maximum diastolic blood pressure (mm Hg), mean +/− SD	111+/− 17.9	112+/− 19.3
Postpartum depression	115 (14.3)	336 (12.5)
**Birth beyond 20 weeks, 1st HDP Pregnancy**		
Birthweight (g), mean +/−SD^b^	2198.9 +/− 1003.1	2142.6+/− 995.6
Gestational age at delivery (weeks), mean +/−SD	34.1 +/− 4.7	33.8 +/− 4.6
Cesarean delivery	526 (63.3)	1517 (54.3)
Male fetal sex	415 (49.9)	1388 (49.6)
Female fetal sex	391 (47.1)	1359 (48.6)
Fetal/infant demise	90 (10.8)	318 (11.4)
Baby admitted to the NICU	424 (51.0)	1294 (46.3)
Length of stay in NICU (days), mean +/−SD	31.2 +/− 34.3	32.0 +/− 37.8

^a^*n* = 457 participants and *n* = 1260 non-respondents. ^b^*n* = 796 participants and *n* = 2686 non-respondents. In some cases (*n*=7) the maximum reported SBP and DBP were not biologically plausible values, thus maximum SBP was restricted to between 60-270 mmHg and maximum DBP was restricted to 40-180 mmHg

All the experiment protocol for involving humans was in accordance to guidelines of the Declaration of Helsinki. Study protocols regarding human subjects were approved by the Advarra Institutional Review Board (IRB), a private independent IRB (https://www.advarra.com/).

### Analysis

Responses were reported descriptively for the sample as a whole, and a preliminary investigation of the role of experience in shaping women’s views was conducted by comparing pre-term and term delivery subgroups, because this distinction is of major importance for clinical care, reliably reported, and both categories are well represented numerically within the study sample. This distinction is of major importance in the emerging literature on prediction and prevention. A skewed distribution of scores was found for the majority of items, so the six-point Likert scales were collapsed into the binary categories ‘Agree’ and ‘Disagree’. The proportion of women agreeing with each statement was compared between subgroups using chi-square. A stringent significance level (< 0.001) was adopted in light of the exploratory nature of the analysis.

The qualitative data provided in the text box was analysed using inductive thematic analysis to explore participants’ responses.

## Results

The final sample consisted of 807 participants from the United States (84%), Canada (5%), United Kingdom (4%) and other countries (7%) (see Table 1).

### Views about predictive tests

Women generally favored these, and there were no differences between subgroups (see Table 2).

**Table 2:** Participants’ views about prediction tests (All comparisons non-significant)

Question	Response	Preterm (***n*** = 545)		Term (***n*** = 262)	
		n	%	n	%
1. Even if a prediction test were not 100% accurate, I would want to take a test early in my pregnancy that lets me know my chances of developing a problem like preeclampsia.	Agree	515	94.5	243	92.7
	Disagree	30	5.5	19	7.3
2. If a simple blood test that predicted if I would get preeclampsia had been available during my previous pregnancy(ies), we probably would have made different choices in the management of my pregnancy.	Agree	444	81.5	202	77.1
	Disagree	101	18.5	60	22.9
3. If a prediction test were available that would tell me in my first trimester that I will most likely get preeclampsia, I would consider terminating my pregnancy.	Agree	30	5.5	10	3.8
	Disagree	515	94.5	252	96.2
4. If a prediction test showed my risk to develop preeclampsia was low, I would be more relaxed about my prenatal care.	Agree	370	67.9	180	68.7
	Disagree	175	32.1	82	31.3
5. A test to predict if I might get preeclampsia at a later stage during pregnancy would give me some peace of mind.	Agree	445	81.7	217	82.
	Disagree	100	18.3	45	17.2
6. A test to predict if I might get preeclampsia at a later stage during pregnancy would add to my anxiety.	Agree	301	55.2	143	54.6
	Disagree	244	44.8	119	45.4
7. A test to predict if I might get preeclampsia at a later stage during pregnancy would be useful to me even though we don’t have a “cure” for preeclampsia.	Agree	526	96.5	253	96.6
	Disagree	19	3.5	9	3.4
8. An accurate prediction test that tells me if I will get preeclampsia is so important that even if it was not included in my healthcare coverage, I would be willing to pay a modest amount out of pocket for it.	Agree	488	89.5	222	84.7
	Disagree	57	10.5	40	15.3

Most participants wanted to use a predictive test in early pregnancy, even if it were not 100% accurate (94%), and a similar proportion (95%) disagreed with wanting a predictive test to consider early termination. A clear majority agreed that the availability of a test would have led to different choices in pregnancy management (79%). On anxiety, most agreed that a prediction of low risk would enable them to be more relaxed about their prenatal care (69%) though not inclined to skip appointments, and a greater proportion thought that a predictive test would give them some peace of mind (81%); however, when asked directly if a predictive test would add to their anxiety, about half (55%) agreed that it would. Most agreed a predictive test would be useful even though ‘we don’t have a ‘cure’ for preeclampsia’ (97%), and that they would be willing to pay a modest fee for an accurate predictive test (88%).

Participants’ qualitative responses show that they value predictive tests because a high-risk result could enable earlier access to care:

*“There is a desperate need for more testing for Preeclampsia. Doctors say they know the signs, yet most women who have experienced very severe cases have been managed poorly.” (Preterm)*

Some participants thought that an inaccurate low-risk result could lead to care delays:

*“…a prediction test that shows a low risk might make some doctors delay treatment of Pre-eclampsia, …because a "test" claimed the risk was low.” (Preterm)*

### Views about diagnostic tests

Views about the value of diagnostic tests were generally positive and did not vary between subgroups (see Table 3).

**Table 3:** Participants’ views about diagnostic tests (All comparisons non-significant)

Question	Response	Preterm (***n*** = 545)		Term (***n*** = 262)	
		n	%	n	%
9. Current methods for diagnosing preeclampsia are sufficient.	Agree	133	24.4	57	21.8
	Disagree	412	75.6	205	78.2
10. A test to diagnose preeclampsia during pregnancy would give me some peace of mind.	Agree	516	94.7	250	95.4
	Disagree	29	5.3	12	4.6
11. A test to diagnose preeclampsia during pregnancy would add to my anxiety.	Agree	182	33.4	85	32.4
	Disagree	363	66.6	177	67.6
12. A test to diagnose preeclampsia during pregnancy would be useful to me even though we don’t have a “cure” for preeclampsia.	Agree	538	98.7	259	98.9
	Disagree	7	1.3	3	1.1
13. An accurate diagnostic test that tells me if I have preeclampsia is so important that even if it was not included in my healthcare coverage, I would be willing to pay a modest amount out of pocket for it.	Agree	500	91.7	245	93.5
	Disagree	45	8.3	17	6.5

Most participants (77%) disagreed that current diagnostic methods are sufficient. A greater proportion agreed that a diagnostic test would give them some peace of mind (95%), be useful to them even though there is no cure (99%), and was so important that they would be willing to pay a modest fee (92%). As with predictive tests, opinion was more divided as to whether a diagnostic test would add to anxiety, but the majority said that it would not (67%).

Qualitative responses suggest participants believed current diagnostic procedures were inadequate and diagnostic tests would be valued because standardized results could prompt speedy, accurate management:

*“There needs to be a common, consistent, universal tool …I developed preeclampsia both times, but they had different requirements on when it was truly "preeclampsia" and not pregnancy induced hypertension.” (Preterm)*

### Views about preventive treatment

On this topic, participants who had had a preterm delivery held significantly different views from the term delivery subgroup, on three of the seven items (see Table 4).

**Table 4:** Participants’ views about treatment for HDPs

Question	Response	Preterm (***n*** = 545)		Term (***n*** = 262)		***p***-value
		n	%	n	%	
14. I would feel nervous taking one baby aspirin per day to reduce my risk of developing preeclampsia without being sure that there were no effects on my baby many years later.	Agree	175	32.1	119	45.4	.0002
	Disagree	370	67.9	143	54.6	
15. Even though there are no studies to prove that a woman’s diet is related to getting preeclampsia, I would be willing to significantly change my diet to try to reduce my risk.	Agree	506	92.	243	92.7	ns
	Disagree	39	7.2	19	7.3	
16. I would be willing to consider other treatments with possible side effects in order to reduce my chance of getting preeclampsia.	Agree	450	82.6	205	78.2	ns
	Disagree	95	17.4	57	21.8	
17. Because baby aspirin has been shown in some studies to safely decrease some women’s risk of developing preeclampsia, I would be willing to take it throughout pregnancy even if it may not help me at all.	Agree	516	94.7	228	87.0	.00015
	Disagree	29	5.3	34	13.0	
18. I would be willing to participate in a research study to test a medication that has been shown to be safe for the baby, but has not yet been used by pregnant women, if it may help prevent preeclampsia.	Agree	433	79.4	187	71.4	ns
	Disagree	112	20.6	75	28.6	
19. When thinking about experimental treatments to prevent preeclampsia, I am more worried about the possibility of getting preeclampsia than I am about the risks of those treatments to my health.	Agree	365	67.0	144	55.0	.0009
	Disagree	180	33.0	118	45.0	
20. When thinking about possible treatments to improve my pregnancy outcomes, I am more concerned with the safety of the treatment to my unborn baby’s health than I am to my health.	Agree	500	91.7	236	90.1	ns
	Disagree	45	8.3	26	9.9	

Participants, especially in the preterm subgroup, disagreed with feeling nervous about taking daily LDA to reduce their risk of developing preeclampsia without certainty that there were no developmental effects on the fetus (64%). Overall, the majority of participants agreed they would be willing to take LDA throughout pregnancy because it has been shown in some studies to safely decrease some women’s risk (92%), with willingness significantly higher in the preterm subgroup.

Most participants would be willing to participate in a research study to test medication that has been shown to be safe for infants, but has not yet been used by pregnant women, if it may prevent preeclampsia (77%). Also, most participants agreed they would be more concerned with the safety of any treatment to their fetus’s health than their own health (91%).

Most participants would be willing to consider treatments with side effects to reduce their preeclampsia risk (82%) and to change their diet, even though there are no studies to prove that diet affects risk (92%). However, when asked to compare risks to themselves from experimental treatments, views were more divided and the strength of opinion differed between the subgroups: overall most participants would be more worried about preeclampsia than about the risks of those treatments to their health (60%), and that proportion was significantly higher in the preterm delivery subgroup (Table 4).

Some participants expressed concerns about lack of information on the risks of LDA:

*“I'm not sure I would [take aspirin] during pregnancy unless the data showed that it was safe.” (Term)*

### Views about expectant management

There were no differences between the subgroups in their views about expectant management (Table 5), although opinion was divided within groups.

**Table 5:** Participants’ views about expectant management (both comparisons non-significant)

Question	Response	Preterm (***n*** = 545)		Term (***n*** = 262)	
		n	%	n	%
21. If I am diagnosed with severe preeclampsia, I am uncomfortable with doctors trying to evaluate when to deliver the baby to “buy time.” It is better to deliver as soon as possible.	Agree	169	31.0	103	39.3
	Disagree	376	69.0	159	60.7
22. If I reached a point where I had to choose, I would rather see how long I could stay pregnant – even if this causes me to face some risks to my own health – rather than risk my baby being born too early.	Agree	401	73.6	187	71.4
	Disagree	144	26.4	75	28.6

The majority of participants opted to put the baby’s interests first, although this varied between considering that it was better to deliver as soon as possible (66%), and aiming to stay pregnant as long as possible for the perceived benefits to the baby (73%).

In their qualitative responses, most participants’ concerns related to the challenge of decision-making about *delivery timing*, given the implications for health and/or survival of mother and baby:

"*Trying to balance my own health needs with those of my unborn baby was the most trying part... The mommy guilt was, and still is, incredible... but so was my desire to stay alive.* " (Preterm)

Participants explained they would delay delivery to increase their baby’s chance of survival, even if this resulted in “serious implications” for their own health. However, many participants also understood that delaying delivery meant increased risks for the baby:

"*My baby was not delivered early enough and he was stillborn, so I lean toward delivery ASAP but not for my own health… to avoid losing another child.*" (Preterm)

Views about delivery timing were also related to participants’ desire to minimize the baby’s time in a neonatal intensive care unit:

"*Every day in the womb exponentially reduces the time an infant spends in the NICU (neonatal intensive care unit).*" (Preterm)

Other participants expressed a preference for early delivery because of the adverse implications of delay for the mother’s health:

"*…There are too many terrible complications that can result after birth for the mother… early delivery is vital.*" (Term)

Participants explained that the decision was also about the risk of leaving their children motherless:

"*I am not willing to die to give my baby extra time and leave him or her, my husband, and other children alone. … if death is on the table, I would choose to save mine…*" (Term)

Participants clarified the difficulty in choosing between their own and their baby’s survival, and the need to support women to prioritize their own health:

"*…Special attention and advocacy needs to be paid to the mother because she will never worry about herself first before her baby unless she has adequate information that her own life could be at risk.*" (Preterm)

Many participants expressed their trust in doctors to support decision-making based on their expertise:

*"…I'd want to defer a great deal to my doctor's guidance, because frankly they have medical training and I do not." (Term)*

However, other participants lacked confidence in doctors because of their experiences and perceptions of incompetence in recognizing and managing the condition:

"*Doctors say they know the signs, yet most women who have experienced very severe cases have been managed poorly. I have very little confidence in the doctors who managed me as a patient*." (Preterm)

## Discussion

Participants had high levels of interest in predictive and diagnostic tests, irrespective of personal experience of a preterm delivery. Participants acknowledged the positive aspects of such testing, valuing knowledge of enhanced risk to improve pregnancy management, with very few who would undertake testing to consider termination. Participants also favored LDA for the prevention of HDPs, and the preterm subgroup particularly supported participation in research into therapeutics. Although women with preterm pre-eclampsia may have had higher rates of maternal and perinatal complications compared to those with term deliveries, responses to the questionnaire were similar from both groups of women across the majority of responses, suggesting that the presence of the disease is a bigger determinant than gestation at delivery. Overall, participants had varied views about expectant management and planned delivery, and there was no single preferred course of action. Our findings suggest that women want better predictive and diagnostic tests, and that their acceptance of risk is likely to vary depending on the severity of their experiences of HDPs.

Improved predictive and diagnostic tests can enable planned delivery, reducing maternal morbidity [17]. Similar to other studies, participants valued predictive and screening tests because of earlier access to care [18, 19]. Support for informed decisions should include information about how such tests relate to better expectant management and improved pregnancy outcomes [20]. While approximately half of the participants believed a predictive test would add to their anxiety, women’s potential anxiety may be less notable in clinical practice than our results suggest since their other perspectives suggest high support for predictive tests, and arguably benefits will outweigh risks when women are well-supported.

Similar to other studies [21], our participants favored preventive measures to reduce risk, including use of LDA - particularly those in the preterm subgroup. For preeclampsia prevention, LDA is currently the only drug for which there is evidence of benefit [22]. Given our participants’ acceptance of preventive aspirin and the supporting clinical literature, HDP screening programs should include LDA for women screening positive, with information on the implication of this treatment while still emphasizing continued vigilance for signs and symptoms of HDP [23]. Participants’ concerns about the implications of treatment on their fetus highlight the need for adequate information to facilitate informed consent [21].

Participants’ views varied most about delivery timing. This may be due to their differing experiences of HDP, perceived implications for other family members, and understanding of the implications of different options. Women trade off possible benefits and harms, doing so in uncertain circumstances. There is some research on women’s experience of preeclampsia [24], but little on how they make decisions about care. A shared decision-making tool for HDPs could enable women to make informed decisions in better accordance with their own values. Women’s views are integral to improving maternity care; the development of such a tool requires further exploration of women’s experiences, expectations and values using more in-depth qualitative methodologies [24, 25].

A predictive test for preeclampsia must be offered early in pregnancy to all women. The literature shows that risk stratification based on maternal factors and pregnancy history is insufficient. The efficacy of LDA has been shown to be greater if treatment is begun early. Knowing that taking LDA is likely to be beneficial because the current pregnancy is at elevated risk may increase women’s willingness to follow a drug regimen.

Any benefits of treatment are less clear for women not classed at higher risk, and there may be downsides to classification: people identified as ‘low risk’ may be less prepared to take precautionary steps or to be vigilant for signs and symptoms of HDP, and, since no screening test is perfect, false reassurance of women and care providers will need to be guarded against.

Once diagnosed, women need to be given up-to-date information on the risks of different courses of action, and subsequent management must take account of the differing preferences expressed. Since these circumstances are often highly charged, urgent medical crises, education and decision-making tools must be deliberately kept straightforward.

The information needs of the general pregnant population about tests for HDPs require further investigation, as does the best way of communicating ‘elevated risk’. Better understanding is needed of how various factors (demographics, pregnancy and neonatal unit experience, etc.) influence attitudes to care.

More representative samples and studies using different methods to assess attitudes would be desirable, including the effects of different phrasing and the ways that trade-offs are presented. The relevance and role of anxiety in screening and treatment decisions is unclear [26, 27].

Women already weigh complex, morally-charged ‘competing risks’. The use of a decision aid for those facing dilemmas needs investigation, based on a current understanding of women’s information needs and of factors affecting test uptake and treatment compliance in different subgroups.

Future comparisons between ‘screen-and-treat’ strategies and other approaches to reducing the incidence and harms of HDPs need to integrate the role of women’s beliefs, attitudes and behavior as mediators of prevention and treatment.

### Strengths and limitations

A strength of our study is that it demonstrates women’s important but neglected role in the research on pre-eclampsia. Limitations include that the convenience sample from the Preeclampsia Registry is not representative of the wider population of pregnant women, and the response rate (22%) will also have introduced bias. Further studies are needed on, samples which include participants from previously under-represented groups. Also, similar research in a normotensive population could have enhanced the findings, but recruiting such participants would have been challenging because of their lack of knowledge or motivation to support research for an unfamiliar condition.

The survey provided clear, consistent evidence of consensus on some topics, e.g., the value of predictive/diagnostic tests, and diversity of views on others, especially those entailing the weighing of different risks. On some topics the wording of questions seemed to influence levels of agreement, but it is unclear whether this reflected genuinely nuanced views, or other differences in question content or wording. Only a minority of questions, all on the topic of prevention, led to differences of opinion between preterm or term subgroups.

The exploratory statistical analysis was confined to a set of simple comparisons. A more comprehensive modelling exercise could make use of the finer-grained attitude information captured in the Likert scales and the additional details in the Preeclampsia Registry dataset.

### Conclusion

Predictive and diagnostic tests and treatments would be valued by women with experience of HDPs. However, varied views about management suggest the need for shared decision-making and educational tools.

## Supplementary Information

**Additional file 1.**

## Data Availability

The datasets used and/or analysed during the current study are available from the corresponding author on reasonable request.

## Abbreviations

HDPs: Hypertensive disorders in pregnancy
LDA: Low-dose aspirin
