# Supplementary material for: Women’s attitudes, beliefs and values about tests, and management for hypertensive disorders of pregnancy
Source: BMC Pregnancy Childbirth. 2021 Sep 30;21:665. doi: 10.1186/s12884-021-04144-2 (PMC8485426; doi:10.1186/s12884-021-04144-2)
Supplement: Supplementary file 1 — Additional file 1. [file 12884_2021_4144_MOESM1_ESM.docx]

**Introduction**

**Your Point of View: Prevention, Diagnosis and Treatment**

The following survey has been added to The Preeclampsia Registry to learn patients' thoughts about preeclampsia tests and treatments, most of which are not yet available to patients. However, as they are being developed and evaluated, it is important for scientists and doctors to understand your point of view regarding these potential tests and treatments. The information will also be used by the Preeclampsia Foundation to advocate for the type of research and clinical interventions that are important to you. This research is being done by the Preeclampsia Foundation, working with several doctors and researchers.

Investigators may also use this information for other research studies. You do not have to answer these questions in order to continue your participation in The Preeclampsia Registry.

**The information you provide in the survey will be de-identified and The Preeclampsia Registry will *not* give anybody your name, contact information, or any information that can identify you. Participating in this survey in *no way* affects your healthcare, insurance, employment or any other status since your personally identifiable information will not be shared.**

This survey will take about 10 minutes to complete. You do **not** need to complete it all at one time. You will be asked questions in four sections. You can read the content at the top of each section for more information about each area.

If you have any questions or concerns, you may contact The Preeclampsia Registry Research Coordinator at (800) 665-9341 or by email at [Registry@preeclampsia.org](mailto:Registry@preeclampsia.org).

Thank you.

“Start Survey”

***Form 2***

**Your Attitudes, Beliefs, and Values**

The following section makes a series of statements that are neither right nor wrong. In each case you should respond with your opinion, indicating if you agree or disagree and how strongly you feel that way.

**Prediction and Diagnostic Testing**

The following statements have to do with the diagnosis of high blood pressure problems in pregnancy and also with newer approaches aimed at predicting such problems before they arise. Problems such as preeclampsia and HELLP syndrome are disorders that only occur during pregnancy or soon after delivery (even up to 6 weeks), but can cause many serious problems for the mother's health. It may also require delivering the baby early to save the mother's and/or baby’s life, or to improve the mother's worsening health. These problems may also cause unborn babies to stop growing. In most of these statements, we use the term "preeclampsia" to include most hypertensive disorders of pregnancy such as eclampsia and HELLP syndrome.

A “*prediction*” test gives an idea on whether a woman has a high or low chance of getting the problem later on during the pregnancy.

A “*diagnostic*” test tells whether a woman has or doesn’t have the problem at the time of the test. It could help a doctor *diagnose* whether or not she really has preeclampsia.

We will first ask you about your feelings on tools used to *predict* preeclampsia.

1) Even if a *prediction* test were not 100% accurate, I would want to take a test early in my pregnancy that lets me know my chances of developing a problem like preeclampsia.

| Strongly Disagree | Disagree | Somewhat Disagree | Somewhat Agree | Agree | Strongly Agree |
| --- | --- | --- | --- | --- | --- |
| 🞆 | 🞆 | 🞆 | 🞆 | 🞆 | 🞆 |

2) If a simple blood test that *predicted* if I would get preeclampsia had been available during my previous pregnancy(ies), we probably would have made different choices in the management of my pregnancy.

| Strongly Disagree | Disagree | Somewhat Disagree | Somewhat Agree | Agree | Strongly Agree |
| --- | --- | --- | --- | --- | --- |
| 🞆 | 🞆 | 🞆 | 🞆 | 🞆 | 🞆 |

3) If a prediction test were available that would tell me in my first trimester that I will most likely get preeclampsia, I would consider terminating my pregnancy.

| Strongly Disagree | Disagree | Somewhat Disagree | Somewhat Agree | Agree | Strongly Agree |
| --- | --- | --- | --- | --- | --- |
| 🞆 | 🞆 | 🞆 | 🞆 | 🞆 | 🞆 |

4) If a *prediction* test showed my risk to develop preeclampsia was low, I would be more relaxed about my prenatal care.

| Strongly Disagree | Disagree | Somewhat Disagree | Somewhat Agree | Agree | Strongly Agree |
| --- | --- | --- | --- | --- | --- |
| 🞆 | 🞆 | 🞆 | 🞆 | 🞆 | 🞆 |

5) A test to *predict* if I might get preeclampsia at a later stage during pregnancy would give me some peace of mind.

| Strongly Disagree | Disagree | Somewhat Disagree | Somewhat Agree | Agree | Strongly Agree |
| --- | --- | --- | --- | --- | --- |
| 🞆 | 🞆 | 🞆 | 🞆 | 🞆 | 🞆 |

6) A test to *predict* if I might get preeclampsia at a later stage during pregnancy would add to my anxiety.

| Strongly Disagree | Disagree | Somewhat Disagree | Somewhat Agree | Agree | Strongly Agree |
| --- | --- | --- | --- | --- | --- |
| 🞆 | 🞆 | 🞆 | 🞆 | 🞆 | 🞆 |

7) A test to *predict* if I might get preeclampsia at a later stage during pregnancy would be useful to me even though we don’t have a “cure” for preeclampsia.

| Strongly Disagree | Disagree | Somewhat Disagree | Somewhat Agree | Agree | Strongly Agree |
| --- | --- | --- | --- | --- | --- |
| 🞆 | 🞆 | 🞆 | 🞆 | 🞆 | 🞆 |

8) An accurate *prediction* test that tells me if I will get preeclampsia is so important that even if it was not included in my healthcare coverage, I would be willing to pay a modest amount out of pocket for it.

| Strongly Disagree | Disagree | Somewhat Disagree | Somewhat Agree | Agree | Strongly Agree |
| --- | --- | --- | --- | --- | --- |
| 🞆 | 🞆 | 🞆 | 🞆 | 🞆 | 🞆 |

Form 3

We will now ask you questions related to tests used to *diagnose* high blood pressure problems in pregnancy.

9) Current methods for *diagnosing* preeclampsia are sufficient.

| Strongly Disagree | Disagree | Somewhat Disagree | Somewhat Agree | Agree | Strongly Agree |
| --- | --- | --- | --- | --- | --- |
| 🞆 | 🞆 | 🞆 | 🞆 | 🞆 | 🞆 |

10) A test to *diagnose* preeclampsia during pregnancy would give me some peace of mind.

| Strongly Disagree | Disagree | Somewhat Disagree | Somewhat Agree | Agree | Strongly Agree |
| --- | --- | --- | --- | --- | --- |
| 🞆 | 🞆 | 🞆 | 🞆 | 🞆 | 🞆 |

11) A test to *diagnose* preeclampsia during pregnancy would add to my anxiety.

| Strongly Disagree | Disagree | Somewhat Disagree | Somewhat Agree | Agree | Strongly Agree |
| --- | --- | --- | --- | --- | --- |
| 🞆 | 🞆 | 🞆 | 🞆 | 🞆 | 🞆 |

12) A test to *diagnose* preeclampsia during pregnancy would be useful to me even though we don’t have a “cure” for preeclampsia.

| Strongly Disagree | Disagree | Somewhat Disagree | Somewhat Agree | Agree | Strongly Agree |
| --- | --- | --- | --- | --- | --- |
| 🞆 | 🞆 | 🞆 | 🞆 | 🞆 | 🞆 |

13) An accurate *diagnostic* test that tells me if I have preeclampsia is so important that even if it was not included in my healthcare coverage, I would be willing to pay a modest amount out of pocket for it.

| Strongly Disagree | Disagree | Somewhat Disagree | Somewhat Agree | Agree | Strongly Agree |
| --- | --- | --- | --- | --- | --- |
| 🞆 | 🞆 | 🞆 | 🞆 | 🞆 | 🞆 |

“Back” “Save For Later” “Next”

***Form 4***

**Treatments**

There is research underway to evaluate different treatments *during pregnancy* to see if these would reduce a woman's chance of getting preeclampsia. The following statements deal with these possible treatments. Please answer as though you were considering another pregnancy, even if you are not. Please select if you agree or disagree and how strongly you feel that way.

14) I would feel nervous taking one baby aspirin per day to reduce my risk of developing preeclampsia without being sure that there were no effects on my baby many years later.

| Strongly Disagree | Disagree | Somewhat Disagree | Somewhat Agree | Agree | Strongly Agree |
| --- | --- | --- | --- | --- | --- |
| 🞆 | 🞆 | 🞆 | 🞆 | 🞆 | 🞆 |

15) Even though there are no studies to prove that a woman's diet is related to getting preeclampsia, I would be willing to significantly change my diet to try to reduce my risk.

| Strongly Disagree | Disagree | Somewhat Disagree | Somewhat Agree | Agree | Strongly Agree |
| --- | --- | --- | --- | --- | --- |
| 🞆 | 🞆 | 🞆 | 🞆 | 🞆 | 🞆 |

16) I would be willing to consider other treatments with possible side effects in order to reduce my chance of getting preeclampsia.

| Strongly Disagree | Disagree | Somewhat Disagree | Somewhat Agree | Agree | Strongly Agree |
| --- | --- | --- | --- | --- | --- |
| 🞆 | 🞆 | 🞆 | 🞆 | 🞆 | 🞆 |

17) Because baby aspirin has been shown in some studies to safely decrease some women's risk of developing preeclampsia, I would be willing to take it throughout pregnancy even if it may not help me at all.

| Strongly Disagree | Disagree | Somewhat Disagree | Somewhat Agree | Agree | Strongly Agree |
| --- | --- | --- | --- | --- | --- |
| 🞆 | 🞆 | 🞆 | 🞆 | 🞆 | 🞆 |

18) I would be willing to participate in a research study to test a medication that has been shown to be safe for the baby, but has not yet been used by pregnant women, if it may help prevent preeclampsia.

| Strongly Disagree | Disagree | Somewhat Disagree | Somewhat Agree | Agree | Strongly Agree |
| --- | --- | --- | --- | --- | --- |
| 🞆 | 🞆 | 🞆 | 🞆 | 🞆 | 🞆 |

19) When thinking about experimental treatments to prevent preeclampsia, I am more worried about the possibility of getting preeclampsia than I am about the risks of those treatments to *my* health.

| Strongly Disagree | Disagree | Somewhat Disagree | Somewhat Agree | Agree | Strongly Agree |
| --- | --- | --- | --- | --- | --- |
| 🞆 | 🞆 | 🞆 | 🞆 | 🞆 | 🞆 |

20) When thinking about possible treatments to improve my pregnancy outcomes, I am more concerned with the safety of the treatment to my *unborn baby’s* health than I am to my health.

| Strongly Disagree | Disagree | Somewhat Disagree | Somewhat Agree | Agree | Strongly Agree |
| --- | --- | --- | --- | --- | --- |
| 🞆 | 🞆 | 🞆 | 🞆 | 🞆 | 🞆 |

“Back” “Save For Later” “Next”

***Form 5***

**Expectant Management – Balancing Mother’s and Baby’s Health**

“Expectant management” is the practice of monitoring a woman who has been diagnosed with preeclampsia to extend her pregnancy as long as safely possible. The hope is to avoid immediate delivery in order to buy more time for the unborn baby to mature. Expectant management may be used with women who are less than 37 weeks pregnant.

These last statements ask you to consider the balance between a mother’s health and her baby’s health, both of which must be considered in expectant management. Please select if you agree or disagree and how strongly you feel that way.

21) If I am diagnosed with severe preeclampsia, I am uncomfortable with doctors trying to evaluate when to deliver the baby to "buy time." It is better to deliver as soon as possible.

| Strongly Disagree | Disagree | Somewhat Disagree | Somewhat Agree | Agree | Strongly Agree |
| --- | --- | --- | --- | --- | --- |
| 🞆 | 🞆 | 🞆 | 🞆 | 🞆 | 🞆 |

22) If I reached a point where I had to choose, I would rather see how long I could stay pregnant – even if this causes me to face some risks to my own health – rather than risk my baby being born too early.

| Strongly Disagree | Disagree | Somewhat Disagree | Somewhat Agree | Agree | Strongly Agree |
| --- | --- | --- | --- | --- | --- |
| 🞆 | 🞆 | 🞆 | 🞆 | 🞆 | 🞆 |

23) Comments: *(open text field)*

“Back” “Save for Later” “Next ”

***Form 6***

**Demographics**

The following questions may help us to understand the role of religious beliefs in your responses to the other questions in this survey. If you prefer not to answer these questions, please just select “I would rather not answer this”.

24) Would you describe yourself as…

*(checkboxes, one response)*

🞎 Extremely religious

🞎 Very religious

🞎 Somewhat religious

🞎 Somewhat non-religious

🞎 Very non-religious

🞎 Extremely non-religious

🞎 I would rather not answer this

🞎 I don’t know

25) How important are religion and religious beliefs to you, now? Are they…

*(checkboxes, one response)*

🞎 Very important

🞎 Fairly important

🞎 Not very important

🞎 Not important at all

🞎 I would rather not answer this

🞎 I don’t know

“Back” “Submit my Survey”

***Form 7***

Thank you for contributing valuable information to The Preeclampsia Registry. Your answers help further our understanding of patient values and beliefs.

While you’re here, please take a few minutes to provide a quick update on your health!

“Provide an Update” “My Registry”
